# Expression/Activation of PAR-1 in Airway Epithelial Cells of COPD Patients: Ex Vivo/In Vitro Study

**DOI:** 10.3390/ijms221910703

**Published:** 2021-10-02

**Authors:** Angela Marina Montalbano, Giuseppina Chiappara, Giusy Daniela Albano, Maria Ferraro, Caterina Di Sano, Patrizio Vitulo, Loredana Pipitone, Fabio Luigi Massimo Ricciardolo, Giulia Anzalone, Mirella Profita

**Affiliations:** 1 Institute for Biomedical Research and Innovation (IRIB), National Research Council of Italy (CNR), 90146 Palermo, Italy; angelamarina.montalbano@irib.cnr.it (A.M.M.); giuseppina.chiappara@irib.cnr.it (G.C.); giusydaniela.albano@irib.cnr.it (G.D.A.); maria.ferraro@irib.cnr.it (M.F.); caterina.disano@irib.cnr.it (C.D.S.); giulia.anzalone@irib.cnr.it (G.A.); 2 Istituto Mediterraneo per i Trapianti e Terapie ad Alta Specializzazione (ISMETT), 90127 Palermo, Italy; pvitulo@ismett.edu (P.V.); lpipitone@ismett.edu (L.P.); 3 Department of Clinical and Biological Sciences, University of Turin, 10124 Turin, Italy; fabioluigimassimo.ricciardolo@unito.it

**Keywords:** protease-activated receptor-1, inflammation, epithelial cells, cigarette smoke, COPD

## Abstract

The role of PAR-1 expression and activation was described in epithelial cells from the central and distal airways of COPD patients using an ex vivo/in vitro model. PAR-1 immunoreactivity was studied in epithelial cells from surgical specimens of the central and distal airways of COPD patients and healthy control (HC). Furthermore, PAR-1 expression and activation were measured in both the human bronchial epithelial cell line (16HBE) and normal human bronchial epithelial cells (NHBEs) exposed to cigarette smoke extract (CSE) (10%) or thrombin. Finally, cell proliferation, apoptosis, and IL-8 release were detected in stimulated NHBEs. We identified higher levels of PAR-1 expression/activation in epithelial cells from the central airways of COPD patients than in HC. Active PAR-1 increased in epithelial cells from central and distal airways of COPD, with higher levels in COPD smokers (correlated with pack-years) than in COPD ex-smokers. 16HBE and NHBEs exposed to CSE or thrombin showed increased levels of active PAR-1 (localized in the cytoplasm) than baseline conditions, while NHBEs treated with thrombin or CSE showed increased levels of IL-8 proteins, with an additional effect when used in combination. Smoking habits generate the upregulation of PAR-1 expression/activation in airway epithelial cells, and promoting IL-8 release might affect the recruitment of infiltrating cells in the airways of COPD patients.

## 1. Introduction

Cigarette smoke is the main risk factor for the development of chronic obstructive pulmonary disease (COPD). It is the third-leading cause of death from disease worldwide in 2020 [1]. The hallmarks of COPD are inflammation of the peripheral airways and the destruction of lung parenchyma or emphysema, with the functional consequence of a poorly reversible airflow limitation. COPD patients showed pathological changes in the central airways and pulmonary arteries as a result of cigarette smoke [2]. COPD is usually sustained by an influx into the lungs of inflammatory cells, which establish complex and highly dynamic interactions with resident cells, such as epithelial cells [3,4].

The epithelial barrier of the airways protects against inhaled agents by maintaining mucociliary clearance through the secretion of antioxidants, protease inhibitors, and antimicrobial factors. In this manner, it regulates the transfer of solutes into the intracellular space of the lung [5]. After exposure to environmental insults, epithelial cells secrete mediators as chemokines, acting as a chemoattractant for inflammatory cells like neutrophils, macrophages, mast cells, eosinophils, and Th-2 cells in the airways [6,7]. Cigarette smoke damages the airway epithelium and affects epithelial integrity, the effectiveness of repair mechanisms, and the release of inflammatory mediators [8,9,10], promoting epithelial dysfunction and the progression of symptoms in COPD.

Protease-activated receptors (PARs) belong to the G-protein-coupled receptors (GPCRs) sub-family and are activated by a serine protease, often involved in the pathophysiological processes of cytokine and chemokine production, platelet aggregation, and cell proliferation [11,12]. Activated PARs rapidly internalize and are targeted to lysosomal degradation after the proteolytic cleavage of their N-terminal extracellular domain by proteases (e.g., thrombin, trypsin, plasmin and others) [13,14]. The recovery of internalized PARs requires either mobilization from the intracellular pool or the synthesis of new receptors [14]; the new N-terminal-tethered ligand domain becomes exposed to the plasma membrane, ready to be activated by cleavage [13,14]. A substantial fraction of the internalized thrombin receptors is recycled on the cell membranes [15,16]. PARs are abundantly recognized for their roles in the development of inflammation in chronic inflammatory diseases [17,18]. All PARs share the same basic mechanism of activation by thrombin [19] and are overexpressed in the alveolar macrophages from smokers with normal lung function [20]. COPD patients have higher maximum thrombin levels (production rate), although this was not linked to severity or inflammatory mediator expression [21].

The aim of the present study was to evaluate the role of the cigarette-smoking habit on PAR-1 receptor expression and activation in airway epithelium using ex vivo/in vitro studies. Ex vivo, we investigated the immunoreactivity of epithelial cells from surgical specimens of central and distal airways of COPD (smoker and ex-smoker) patients, compared to healthy control subjects. We used two antibodies that recognize the total PAR-1 receptor (ATAP-2) and non-active PAR-1 receptor (H-111) to detect the levels of PAR-1 activation. In vitro, we evaluated the effect of cigarette smoke extract (CSE) on PAR-1 expression and activation in normal human bronchial epithelial cell lines (16HBE), and in primary normal human bronchial epithelial cells (NHBEs). Finally, we studied whether CSE or thrombin (alone or in combination) affected higher levels of cell proliferation, apoptosis, and chemokine release.

## 2. Results

### 2.1. Demographic Characteristics of the Subjects

Demographic characteristics and functional evaluations of the study groups are shown in Table 1. All recruited patient groups were similar in age.

Table 1 Data are shown as mean ± SD. Abbreviations: HC = never-smoked subjects with normal lung function; COPD = patients with chronic obstructive pulmonary disease; FEV1 = forced expiratory volume in 1 s; FVC = forced vital capacity.

### 2.2. Expression of PAR-1 Receptor in Airway Epithelial Cells of COPD Patients

**Figure 1:**
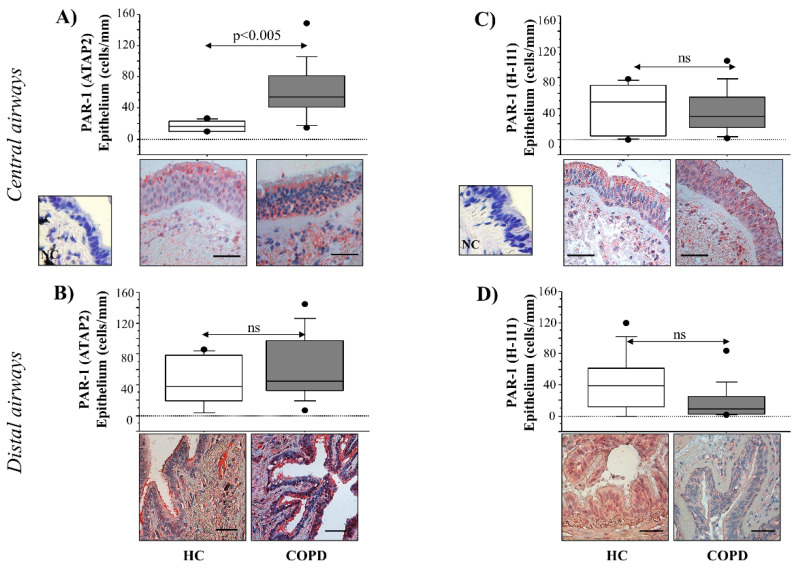
Immunoreactivity of anti-PAR-1 ATAP-2 Ab and anti-PAR-1 H-111 Ab in surgical specimens from central and distal airways of HC (*n* = 10), and COPD (*n* = 15) subjects. (**A**,**B**) Cells were stained with an anti-PAR-1 ATAP-2 Ab and (**C**,**D**) with an anti-PAR-1 H-111 Ab. Negative control was performed using rabbit immunoglobulins (see Section 2 for details). The scale bar is 50 µm. Counts of the number of positive epithelial cells/mm in the basement membrane is shown. Representative Immunostaining at 40× magnification is shown. Results were expressed as box plots representing median and 25–75 percentiles. Statistical analysis was performed with the Kruskal–Wallis test, followed by Fisher’s PLSD correction for multiple comparisons. Significance was accepted at *p* < 0.05.

PAR-1 ATAP-2 (total receptor) and PAR-1 H-111 (no active receptor) immunoreactivity was evaluated in the epithelium from the central and distal airways of HC and COPD subjects by immunohistochemistry. PAR-1 (ATAP-2) immunoreactivity (cells/mm) of epithelial cells showed higher levels in the central (*p* < 0.005) and distal airways (*p* < ns) of COPD patients compared to HC subjects (Figure 1A,B). The PAR-1 H-111 immunoreactivity of epithelial cells described a trend toward lower levels in both central (*p* < ns) and distal (*p* < ns) airways of COPD patients compared to HC subjects (Figure 1C,D).

### 2.3. Total and No Active PAR-1 Distribution in Central and Distal Airways

**Figure 2:**
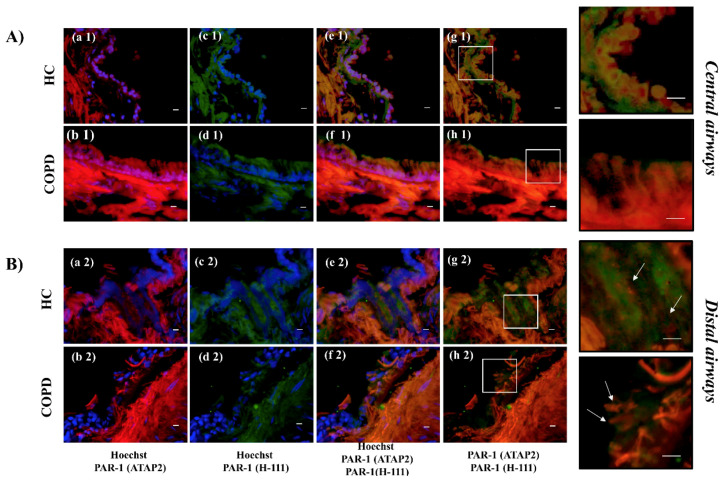
Immunofluorescent reactivity of anti-PAR-1 ATAP-2 A and anti-PAR-1 H-111 Abs in epithelial cells from surgical specimens from the central and distal airway of HC and COPD subjects. (**A**) Epithelial cells from the central airways showed single staining for anti-PAR-1 ATAP-2 Ab (**red**) (**a1**,**b1**) or anti-PAR-1 H-111 Ab (**green**) (**c1**,**d1**), or a double staining for anti-ATAP-2/H-111Abs with (**e1**,**f1**) or without nuclear staining Hoechst (**blue**) (**g1**,**h1**). (**B**) Epithelial cells from distal airways showed the single staining for anti- PAR-1 ATAP-2 Ab (**red**) (**a1**,**b1**) or anti- PAR-1 H-111 Ab (**green**) (**c1**,**d1**), or a double staining for anti- ATAP-2/H-111 with (**e2**,**f2**) or without nuclear staining with Hoechst (**blue**) (**g1**,**h1**). The scale bar is 50 μm. Representative staining for each marker was shown. The magnification area shows the co-localization/distribution of anti-PAR-1 ATAP-2 Ab and anti- PAR-1 H-111 Ab immunofluorescent staining.

The distribution of immunofluorescent staining of anti-PAR-1 ATAP-2 Ab (Total receptor) (red color) was higher in the epithelium from the central airways of COPD patients than in the HC (Figure 2A (a1, b1)). No differences were observed in epithelium from the distal airway of COPD compared to the HC (Figure 2B (c1, d1)). Finally, the distribution of immunofluorescent staining of anti-PAR-1 H-111 (total receptor) (green color) Ab was lower in the central airways and did not show substantial differences in airway epithelial cells from COPD compared to HC subjects (Figure 2A (e1, e2, f1, f2)).

Figure 2A shows the immunofluorescent staining of anti-ATAP2 Ab (red) (a1) and anti-H111 Ab (green) (c1) in epithelial cells from HC subjects. Panel e1 demonstrates the multiple staining of anti-ATAP2 Ab (red) and anti-H111 Ab (green) with the blue staining of nuclei obtained via Hoechst in the epithelial cells from HC. Panel g1 shows the colocalization of anti-ATAP2 (red) and anti-H111 (green) antibodies with unstained nuclei, and its magnification demonstrates anti-H111 Ab (no active receptor) mainly localized in the cell membrane and anti-ATAP2 (red) (total receptor) in the cytoplasm.

Panels b1 and d1 show the immunofluorescent staining of anti-ATAP2 Ab (red) and anti-H111 Ab (green), with the blue staining of nuclei obtained via Hoechst in epithelial cells from the central airways of COPD subjects. Panel f1 shows the colocalization of anti-ATAP2 Ab (red) and anti-H111 Ab (green), with the blue staining of nuclei obtained with Hoechst in the epithelial cells from the central airways of COPD patients. Panel h1 shows the colocalization of ATAP2 (red) and H111 (green) Abs with unstained nuclei. The magnification of panel g1 and h1 demonstrates low immunoreactivity for anti-PAR-1 H111 Ab (green) in the cell membrane, and the red fluorescence of anti-ATAP2 Ab is localized in both the cell membrane and cytoplasm of epithelial cells of central airways with COPD, mainly in the apical pole of columnar cells.

Figure 2B shows the distributions of immunofluorescent staining of anti-ATAP2 Ab (total receptor—red) (a2) and anti-PAR-1 Ab (H-111) (no active receptor—green) (c2) in the distal airways of HC subjects. Panel e2 demonstrates the colocalization of anti-PAR-1 ATAP2 Ab and anti-PAR-1 H111 Ab, with blue staining of the nuclei, and panel g2 describes the colocalization of anti-PAR-1 ATAP2 Ab and anti-PAR-1 H111 Ab, with no staining of the nuclei. The magnification of panel g2 shows the distribution of anti- PAR-1 H111 Ab, mainly in the cell membrane, while anti-PAR-1 ATAP2 Ab is predominantly expressed in the cytoplasm of epithelial cells from the distal airways of HC.

Panels b2 and d2 show the distributions of anti-PAR-1 ATAP2 Ab (red) in the panel (b2) and anti- PAR-1 H111 Ab (green) in the epithelial cells from the distal airways of COPD subjects. Panel f2 demonstrates the colocalization of anti-PAR-1 ATAP2 Ab and anti-PAR-1 H111 Ab with blue staining of the nuclei. Panel h2 shows the colocalization of anti-PAR-1 ATAP2 Ab and anti-PAR-1 H111 without blue staining of nuclei. Panel g 2 and h2 magnifications show the low levels of anti-PAR-1 H111 Ab (green) on the cell membrane, while the red fluorescence of anti-PAR-1 ATAP2 Ab (red) prevails in the cytoplasm of epithelial cells from the distal airways.

### 2.4. PAR-1 Distribution in Central and Distal Airways of COPD (Smokers and Ex-Smokers)

**Figure 3:**
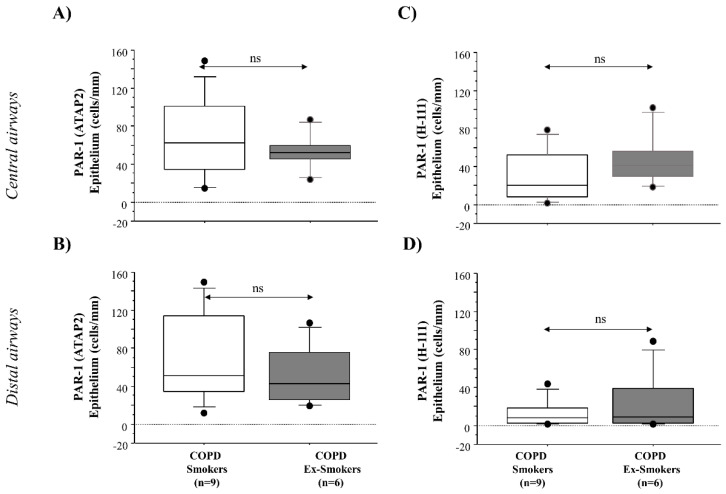
Anti-PAR-1 ATAP-2 Ab, and anti-PAR-1 H-111 Ab immunoreactivity in epithelial cells from surgical specimens from central and distal airways of COPD smokers (*n* = 9), and COPD ex-smokers (*n* = 6). (**A**,**B**) Cells were stained with anti-PAR-1 ATAP-2 Ab; (**C**,**D**) cells were stained with an anti-PAR-1 H-111 Ab. A negative control was tested using rabbit immunoglobulins (see Section 2 for details). The scale bar is 50 µm. Counts for the number of positive epithelial cells/mm in the basement membrane are shown. Representative immunostaining at 40× magnification is shown. Results are expressed as a box plot representing median and 25–75 percentiles. Statistical analysis was performed using the Kruskal–Wallis test followed by Fisher’s PLSD correction for multiple comparisons. Significance was accepted at *p* < 0.05.

PAR-1 ATAP-2 and PAR-1 H-111 immunoreactivity was evaluated in epithelial cells from surgical specimens of both the central and distal airways of COPD ex-smokers, compared to COPD smokers. Epithelial cells from the central and distal airway show a trend toward lower levels of anti-PAR-1 ATAP-2 Ab (*p* < ns) and higher levels of anti-PAR-1 H-111 Ab immunoreactivity in COPD ex-smokers compared to COPD smokers (*p* < ns) (Figure 3).

**Figure 4:**
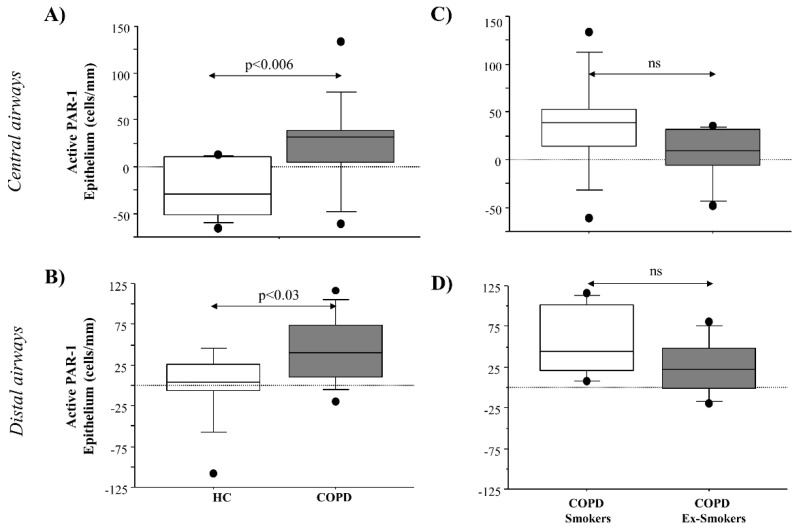
PAR-1 active receptor immunoreactivity in epithelial cells from surgical specimens from epithelial cells of HC and COPD patients. PAR-1 active receptor immunoreactivity in epithelial cells of (**A**) central and (**B**) distal airways of COPD patients (*n* = 15) and HC subjects (*n* = 10), and of (**C**) central and (**D**) distal airways of COPD smokers and ex-smokers. Results shown are the differences between the counts of positive epithelial cells/mm basement membrane obtained with anti-PAR-1 ATAP-2 Ab immunoreactivity and with anti- PAR-1 H-111 Ab immunoreactivity (see Materials and Methods section). Box plots represent the median and 25–75 percentiles of the results. Statistical analysis was performed by Kruskal–Wallis test followed by Fisher’s PLSD correction for multiple comparisons. Significance was accepted at *p* < 0.05.

**Figure 5:**
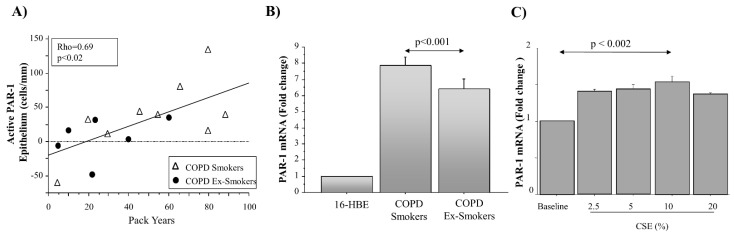
(**A**) Correlations between pack-years and PAR-1 active receptor immunoreactivity in epithelial cells from the central airways of COPD (*n* = 15) patients. Results shown are the differences between the counts of positive epithelial cells/mm basement membrane obtained with anti-PAR-1 ATAP-2 Ab and anti-PAR-1 H-111 Ab (see Materials and Methods section). Data were reported as individual values of COPD ex-smokers and COPD smokers. Statistical analysis was performed with Spearman’s rank test. Significance was set at *p* < 0.05. (**B**) Expression of PAR-1 mRNA in 16HBE cell line and in micro-dissected bronchial epithelium from the central airways of COPD smokers (*n* = 3) and ex-smokers with COPD (*n* = 3) assessed by real-time PCR (see Section 2 for details). (**C**) Effects of different concentrations of CSE (0 to 20%) on PAR-1 m-RNA expression by RT-PCR in 16HBE cells. GAPDH gene expression was used as an endogenous control for normalization. Relative quantitation of mRNA was carried out using the comparative CT method (mean ± SD). For B and C, data are expressed as a fold change above 16HBE cells ± SD. Statistical analysis was performed with an ANOVA test, with Fisher’s correction for multiple comparisons or Student’s *t*-test. Significance was accepted at *p* < 0.05.

Active PAR-1 immunoreactivity was calculated in airway epithelial cells, as previously described (see Materials and Methods). Active PAR-1 was significantly increased in epithelial cells from both the central and distal airways of COPD patients, compared to HC subjects (*p* < 0.006 and *p* < 0.03, respectively) (Figure 4A,B). In addition, active PAR-1 showed lower levels in epithelial cells from both the central and distal airways of COPD ex-smokers than in COPD smokers (Figure 4C,D). Finally, active PAR-1 immunoreactivity of epithelial cells from the central airways of COPD patients (smokers and ex-smokers) was positively correlated with Pack Years (Rho = 0.69) (Figure 5A).

### 2.5. PAR-1 mRNA Levels in Central Airways and 16HBE Stimulated with CSE by RT-PCR

The levels of PAR-1 m-RNA were evaluated in micro-dissected bronchial epithelium from frozen sections of COPD smoker and COPD ex-smoker samples, and in 16HBE cells (used as control sample). The levels of PAR-1 mRNA were higher in micro-dissected epithelium from smokers with COPD (*n* = 3) than in COPD ex-smokers (*n* = 3) (*p* < 0.001) (Figure 5B). The results were expressed as a fold change with respect to the 16HBE values. Finally, the levels of PAR-1 mRNA were increased in 16HBE that were stimulated with different concentrations of CSE (2.5%, 5%, 10% and 20%), reaching significantly higher levels in 16HBE stimulated with CSE at 10%, rather than the baseline conditions of the cells (*p* < 0.002). Results were expressed as a fold change (obtained choosing baseline cells as a reference sample) (Figure 5C).

### 2.6. PAR-1 Activation in Epithelial Cells

**Figure 6:**
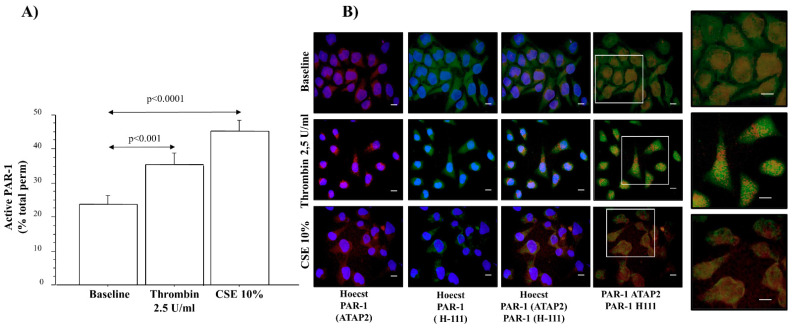
Active PAR-1 in epithelial cell lines. (**A**) 16HBE cells were stimulated with thrombin 2.5 U/mL and CSE 10% to evaluate the activation of PAR-1, using flow cytometric analysis. Bars represent the mean ± S.D. of fluorescence intensity of three separate experiments. The results shown are the differences between the percentage of total counts obtained with anti-PAR-1 ATAP-2 Ab and anti-PAR-1 H-111 Ab (see the Materials and Methods section). Statistical analysis was performed by Student’s *t*-test. Significance was set at *p* < 0.05. (**B**) Anti-PAR-1 ATAP-2 ab, and anti-PAR-1 H-111 ab localization and colocalization/distribution in 16HBE stimulated with thrombin at 2.5 U/mL and CSE 10%, tested by immunofluorescence analysis. The representative immunofluorescence analysis is shown. The scale bar is 50 µm. The magnification shows the area of the co-localization/distribution of anti-PAR-1 ATAP-2 and PAR-1 H-111 Abs immunofluorescent staining.

The levels of anti-PAR-1 ATAP-2 Ab (total receptor) and anti-PAR-1 Ab H-111 (no active receptor) immunoreactivity were observed in an in vitro model of 16HBE cells stimulated with thrombin or CSE at 10%, using flow cytometry and immunofluorescence. Active PAR-1 showed a statistically significant increase in 16HBE cells stimulated with thrombin or CSE than in baseline conditions (Figure 6A).

Immunofluorescence analysis showed the localization of the two antibodies used in our study (Figure 6B). The first column of Figure 6B used anti-PAR-1 ATAP-2 Ab (red) and Hoechst (blue color) to stain nuclei under the different conditions (Figure 6B; panel a1,b1,c1). The second column shows the anti-PAR-1 H-111 Ab (green fluorescence) and Hoechst (blue color of the nuclei), under the three different conditions (Figure 6B panel a2,b2,c2). The third and fourth columns show the double staining of anti-PAR-1 ATAP-2 (red fluorescence) Ab and anti-PAR-1 H-111 (green fluorescence) Ab with (Figure 6B panel a3,b3,c3) and without Hoechst (Figure 6B panel a4,b4,c4). Figure 6B (a3, a4) demonstrate that immunofluorescent anti-PAR-1 ATAP-2 Ab), and anti-PAR-1 H-111 Ab, are localized in the cell membrane in baseline conditions. The stimulation of 16HBE with thrombin 2.5 U/mL (Figure 6B, Panel b3,b4) or CSE 10% (Figure 6B, Panels c3,c4) demonstrate that the localization of anti-PAR-1 ATAP-2 Ab (red color) immunoreactivity increased in the cytoplasm, and anti-PAR-1 H-111 Ab immunoreactivity on the cell membrane (see the magnification area of the panel). The increased amount of PAR-1 in the cytoplasm might be due to an increase in PAR-1 internalization and activation.

**Figure 7:**
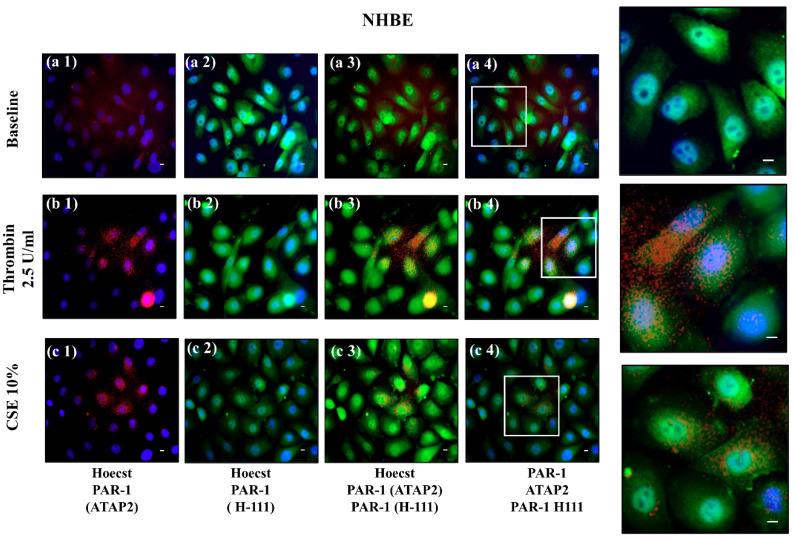
Active PAR-1 in NHBE. Evaluation of anti-PAR-1 ATAP-2 Ab, and anti-PAR-1 H-111 Ab localization and colocalization/distribution in NHBE cells stimulated with thrombin 2.5 U/mL and CSE 10% using immunofluorescence analysis. The stimulated cells were fixed and stained with anti-PAR-1 ATAP-2 Ab (red fluorescence) (**a1**,**b1**,**c1**), and anti-PAR-1 H-111 (green fluorescence) Ab (**a2**,**b2**,**c2**), and subjected to immunofluorescent microscopy. Cell nuclei were stained with Hoechst 33,342 (blue fluorescence). Anti-PAR-1 ATAP-2 and anti-PAR-1 H-111 abs colocalization/distribution was studied with (**a3**,**b3**,**c3**), and without Hoeschst (**a4**,**b4**,**c4**). The scale bar is 50 µm. The magnification shows the co-localization/distribution of anti-PAR-1 ATAP-2 Ab and anti-PAR-1 H-111 Ab immunofluorescent staining. Nonimmune IgG was used as a negative control.

Finally, Figure 7 shows the localization of the immunoreactivity of the Abs in NHBE cells by immunofluorescence. The first column shows anti-PAR-1 ATAP-2 (red fluorescence) Ab and the blue color is used to stain nuclei in different conditions (Figure 7, panel a1,b1,c1). The second column demonstrates anti-PAR-1 H-111 (green fluorescence) Ab and the blue color of the nuclei in the three conditions (Figure 7, Panel a2,b2,c2). The third and fourth columns show the double staining of anti-PAR-1 ATAP-2 (red fluorescence) Ab and anti-PAR-1 H-111 (green fluorescence) Abs with (Figure 6B Panel a3,b3,c3) and without Hoechst (Figure 7 panel a4,b4,c4). Both Abs are localized in the cell membrane, and anti-PAR-1 H-111 (green fluorescence) Ab appears more expressed than anti-PAR-1 ATAP-2 (red fluorescence) Ab (Figure 7 panel a3,a4) in the baseline conditions. NHBEs treated with thrombin 2.5 U/mL or CSE 10% show increased anti-PAR-1 ATAP-2 Ab (red fluorescence) localization in the cytoplasm, and anti-PAR-1 H-111 Ab in the cell membrane (Figure 7, Panels b3,b4,c3,c4,d3).

### 2.7. Apoptosis, Cell Proliferation and IL-8 Release in NHBE Cells Stimulated with Thrombin and CSE

**Figure 8:**
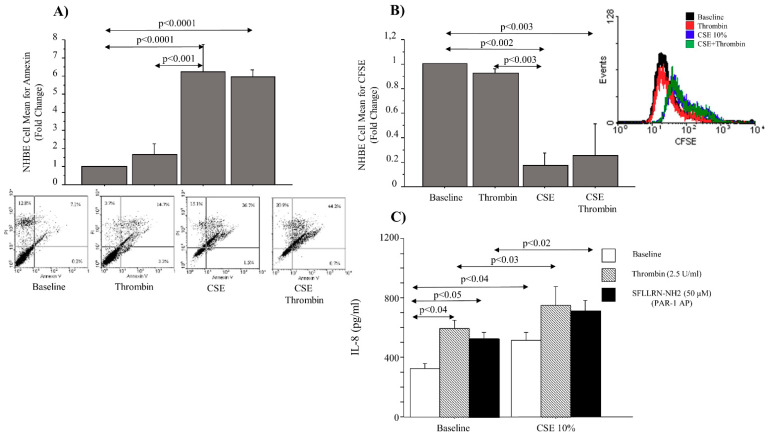
Cell apoptosis, cell proliferation and IL-8 release in NHBEs stimulated with thrombin and CSE. NHBEs, cultured in the presence and absence of thrombin at 2.5 U/mL, alone or in combination with CSE at 10%, were used for evaluating (**A**) apoptosis using an Annexin V Test (see Materials and Methods for details) and (**B**) proliferation using a CFSE test (see Materials and Methods for details). Bars represent the mean ± SD fluorescence intensity (MFI) of three separate experiments and were plotted as fold changes compared to the untreated cells, which were chosen as the reference sample. Representative flow cytometry of cell apoptosis and of cell proliferation are shown (**C**) as IL-8 release in NHBEs stimulated with thrombin 2.5 U/mL or SFLLRN-NH2 (50 μM) (PAR-1 AP) in the presence or absence of CSE at 10%. The values shown are the mean ± SD of three separate experiments. Statistical analysis was performed by ANOVA test followed by Fisher’s PLSD multiple comparison test.

CSE at 10% increased cell apoptosis and reduced cell proliferation in NHBEs, compared to the basal conditions (*p* < 0.0001 and *p* < 0.002, respectively). Thrombin, alone or in combination with CSE at 10%, did not affect cell apoptosis or proliferation in comparison with baseline conditions. Thrombin, in combination with CSE at 10%, did not affect cell apoptosis or proliferation in comparison with cells stimulated with CSE at 10% alone (Figure 8). Thrombin (in the presence or absence of CSE at 10%), did affect cell apoptosis or proliferation, and SFLLRN-NH2 (PAR-1 selective activating peptide) was not used in the experiment design (Figure 8A,B).

Thrombin or SFLLRN-NH2 (50 μM) (PAR-1 AP) induced the release of IL-8 in the supernatants of stimulated NHBEs, compared to the basal conditions (*p* < 0.04; *p* < 0.05, respectively). CSE at 10% increased the release of IL-8 in the supernatants of stimulated NHBEs, compared to basal conditions (*p* < 0.04). Finally, we showed that thrombin or SFLLRN-NH2 (50 μM) (PAR-1 selective activating peptide) increased IL-8 release in the supernatants of NHBEs stimulated with CSE, compared to NHBEs treated with CSE alone (*p* < 0.02; *p* < 0.03, respectively) (Figure 8C).

## 3. Discussion

Together with PAR-1 expression, this study describes for the first time the levels of PAR-1 activation in epithelial cells from the central and distal airways of COPD patients. PAR-1 immunoreactivity was obtained in COPD patients by using two different monoclonal antibodies (ATAP2 and H111), mapping different epitopes of PAR-1, and gave us a useful method to evaluate the different levels of PAR-1 expression and activation in the airways of COPD patients. We showed that the PAR-1 transcript increased in micro-dissected epithelial cells from COPD smokers rather than in COPD ex-smokers and that active PAR-1 closely correlates with cigarette smoking (pack-years) in epithelial cells from the central airways. Furthermore, we described the action of cigarette smoke extract on the transcription of PAR-1 mRNA, using an in vitro model of the human bronchial epithelial cell line 16HBE. All these findings support the potential role of cigarette smoke in PAR-1 synthesis and function in COPD patients. Finally, we described how the higher levels of PAR-1 expression and activation might be involved in the airway inflammation of COPD through IL-8 production, rather than on the mechanisms inherent in tissue damage (apoptosis, proliferation).

The airway epithelium is involved in the mechanism of lung defense as a physical barrier to inhaled substances, including pollutants, allergens, bacteria, viruses, and cigarette smoke. It maintains sterility in the lower airways, promoting the cellular and molecular mechanisms of the innate immune system [22]. The dysregulation of cell function, related to airway epithelial cells, may contribute to the pathogenesis of major lung diseases such as COPD [23]. A wide range of cellular and molecular signaling due to PARs is associated with the pathophysiology of the airway epithelium [24]. PAR-1 activation is obtained by serine proteases (thrombin, trypsin, and mast cell tryptase) [12]. Thrombin affects its target tissues, often via the proteolytic activation of cell surface G-protein-coupled receptors, by the unmasking of an N-terminal amino acid sequence cleaved between Arg41 and Ser42 that acts as a tethered, self-activating ligand [15,16,25,26]. The cleaved receptor is rapidly internalized into lysosomes and then degraded [27]. PAR-1 was diffusely present in the cellular cytoplasm of epithelial cells, mainly located in the apical side of columnar epithelial cells, both in healthy subjects and steroid-treated asthmatic patients [28], and PAR-1 trafficking was observed in epithelial cells from steroid-naive asthmatic patients [28]. All these findings, together with the evidence of higher maximum rates of thrombin production in COPD patients [21], allowed us to evaluate PAR-1 expression and activation in airway epithelial cells from the central and distal airways. We measured PAR-1 immunoreactivity by means of two antibodies: anti-thrombin receptor ATAP2, and anti-thrombin receptor H-111. ATAP-2 Ab recognizes both cleaved and un-cleaved receptor forms (total receptor), and thrombin R H-111 ab recognizes the un-cleaved receptor form (no active receptor). The differences between the immunoreactivity of anti-ATAP2 and anti-H-111 abs identified the levels of active PAR-1. The immunoreactivity of anti-PAR-1 ATAP-2 Ab was higher, while anti-PAR-1 H-111 ab was lower in epithelial cells from COPD subjects, rather than in HC from both the central and distal airways. The differences in the immunoreactivity of the two Abs underlined higher levels of PAR-1 activation in the epithelial cells from the airways of COPD patients, leaving us to suppose the involvement of the receptor in the disease pathogenesis. Immunofluorescent PAR-1 immunoreactivity was demonstrated in central airways: (1) inactive receptor (H111 Ab) (green colors), mainly localized in the cell membrane, and total receptor (ATAP2 Ab) (red colors) in the cytoplasm of epithelial cells of HC; (2) inactive receptor (H111 ab) (green colors), localized in the cell membrane (low levels of intensity) and total receptor (ATAP2 Ab) (red colors), in both the cell membrane and cytoplasm (high levels of intensity), with a specific localization in the apical pole of the columnar epithelial cells from COPD patients. Immunofluorescent PAR-1 immunoreactivity was demonstrated in the distal airways: (1) inactive receptor (H111 Ab) (green colors), localized in the cell membrane, and total receptor (ATAP2 Ab) (red colors) predominantly in the cytoplasm of epithelial cells of HC; (2) low levels of the anti-inactive receptor (H111 Ab) (green colors) in the cell membrane, with a prevalence of the total receptor (ATAP2 ab) (red colors), localized in the cytoplasm of epithelial cells from COPD patients. Our findings support the concept that the reduction of the inactive receptor (H111 Ab) on the cell membrane and the increase of the total receptor (ATAP2 Ab) might be due to the shift of PAR-1 from the cell membrane to the cytoplasm in the airway epithelial cells of COPD patients. These findings suggest the synthesis of new receptors (detected by PAR-1 H-111 Ab), after cleavage/activation and internalization, re-expressed on the cell surface from a pre-formed pool that is present in the cytoplasm. These data might underline PAR-1 trafficking from the cytoplasm to the cell membrane in both the central and distal airway epithelium from COPD patients. Furthermore, the lower levels of immunofluorescent PAR-1 immunoreactivity observed in the distal airways support the concept that the mentioned mechanisms could be stronger in the central airways than in the distal airways.

Serine proteases secreted by neutrophils (i.e., elastase, cathepsin G, and proteinase 3) and mast cells (i.e., tryptase and chymase) are involved in the pathophysiology of airways disease induced by cigarette smoke [29,30,31]. Enzyme and PARs interaction might lead to airway damage, inflammation, and hyper-responsiveness. The overexpression of PAR-1 proteins in AMs from smokers is involved in the pathophysiology of chronic airway disease [20]. We detected higher levels of anti-total PAR-1 (ATAP-2 Ab) immunoreactivity in the central and distal airways of COPD smokers than in COPD ex-smokers. Anti-PAR-1 (H-111 Ab) immunoreactivity showed lower levels in both the central and distal airways of COPD ex-smokers than in COPD smokers. Differences of Ab immunoreactivity gave us a measurement of higher levels of PAR-1 activation in epithelial cells from both the central and distal airways of COPD smokers, rather than in COPD ex-smokers. Furthermore, we observed a positive correlation between the levels of active PAR-1 expression and smoking habit (pack-years) in COPD smokers, and smoking cessation influenced the reduction of the PAR-1 mRNA transcript in the micro-dissected epithelium of frozen sections from the central airway of COPD smokers, rather than from the central airway of COPD ex-smokers. Finally, in vitro experiments showed an increase in the PAR-1 transcript in cells stimulated with CSE. All these findings suggest the pivotal role of cigarette smoking in the expression and activation of PAR-1 in the airways, most probably contributing to the pathogenesis of COPD. Accordingly, 16HBE cells stimulated with thrombin and CSE showed higher levels of PAR-1 activation. The immunofluorescent staining of untreated 16HBE with anti-total receptor (ATAP-2) and anti-inactive receptor (H-111) Abs showed low levels of the total receptor, mainly localized in the cytoplasm, and inactive PAR-1 in the cell membranes. The stimulation of 16HBE with thrombin increased the presence of the total receptor in the cytoplasm, associated with a decrease in the no-active receptor in the cell membrane, suggesting the consequent activation and internalization of PAR-1. The same distribution of anti-PAR-1 ATAP2 Ab (red) and anti-PAR-1 H111 Ab (green) is observed in those cells stimulated with CSE.

The use of NHBEs represents a significant bridge between in vitro and in vivo studies in the experimental research of respiratory diseases [32]. However, NHBEs are difficult to obtain from human tissue, limiting in vitro cell cultures and experimental procedures. Accordingly, we used NHBEs in a limited number of experiments that are useful to support the pathophysiological changes of epithelial cells of the airway associated with the cleavage and activation of PAR-1. We first identified that thrombin or CSE activated PAR-1 in the 16HBE cell line (detection of total PAR-1 and no active PAR-1 by cytofluorimetry and immunofluorescent immunoreactivity), then we obtained the same result in a few experiments with NHBEs (detection of immunofluorescent immunoreactivity of total PAR-1 and of inactive PAR-1). These results supported the concept that both cigarette smoke and thrombin might be involved in PAR-1 activation and trafficking in airway epithelial cells.

Cigarette smoking affects the chemotaxis, proliferation, and remodeling of the extra-cellular matrix, impairing the normal function of epithelial cells derived from human airways [33]. The dysregulation of airway epithelial cell function related to environmental triggers, like cigarette smoke, may contribute to the pathogenesis of major lung diseases such as COPD, and secrete mediators such as IL-8, involved in the recruiting and activating of inflammatory cells [34]. It is known that thrombin production occurs in patients with pulmonary inflammatory diseases and modulates the mechanism of tissue repair, promoting vascular permeability, secretion of proteases, cell adhesion, spread, and the proliferation and secretion of proinflammatory mediators, such as IL-6, IL-8/CXCL8, and PGE2 [35,36]. With the use of NHBEs in our experimental conditions, we observed that CSE, rather than thrombin, affected cell apoptosis and proliferation, and both CSE and thrombin induced the production of cell chemoattractant IL-8 (soluble), with an additional effect when used in combination. Furthermore, the stimulation of NHBEs with SFLLRN-NH2 (PAR-1 selective activating peptide) in the presence or absence of CSE increased the release of IL-8, underlining the contribution of PAR-1 activation. Although our data on PAR-1 expression appear to contradict previous studies obtained in the cardiovascular system [37,38], altogether, our findings identified that smoking habits might generate the upregulation of PAR-1 expression/activation and promote IL-8 release, rather than apoptosis or cell proliferation, in airway epithelial cells. These findings might suggest that PAR-1 activity favors the pathogenic mechanisms of COPD, recruiting infiltrating cells in the airways.

## 4. Materials and Methods

### 4.1. Patient Population

Patients underwent surgery for lung cancer and were recruited at ISMETT-Palermo, Italy. The study was approved by the ISMETT Ethics Committee (Prot. Number 217807, date of approval 30/06/08) and conforms with the Helsinki Declaration. Written informed consents were obtained from each patient. The diagnosis of COPD and the assessment of its severity were defined and classified according to the criteria reported by the Global Initiative for Obstructive Lung Disease (GOLD) guidelines for COPD management (GOLD stage I–II) [39]. The subjects were classified into the following groups: (i) never-smoked subjects without COPD (HC) (*n* = 10); (ii) patients (>20 pack/year) with COPD (*n* = 15), 6 of which were ex-smokers COPD and had stopped smoking more than 1 year before the study began. COPD subjects with exacerbations within 1 month prior to the study were excluded. Subjects had negative skin tests for common allergen extracts and had no history of asthma or allergic rhinitis. COPD patients were treated with bronchodilators and were classified based on preoperative lung function: FEV1 in less than 80% of the reference sample, FEV1/FVC, less than 70%, and bronchodilation effect of less than 12%. The patients were not under corticosteroid therapy (neither inhaled nor systemic) and not under antibiotics, and also did not have exacerbations during the month preceding the study.

### 4.2. Immunohistochemistry

Tissue specimens from tumor-free central bronchi and peripheral lung tissue were selected, fixed with 10% neutral buffer formalin, and embedded in paraffin wax. Sequential sections (3 μm thick) were placed on poly-l-lysine coated slides, deparaffinized in xylene, rehydrated in a descending ethanol series, and stained with hematoxylin and eosin (HE). To reveal the content of the samples, the stain sections were deparaffinized in xylene, rehydrated in a descending ethanol series, and stained with primary antibodies. LSAB2 Dako kit (Code Nu K0674) (Dako, Glostrup, Denmark) and Fuchsine Substrate-Chromogen System Dako [40] were used for the staining. The immunoreactivity of the samples was evaluated using the following antibodies: rabbit polyclonal thrombin R (H-111) antibody: sc-5605 (Santa Cruz Biotechnology, Inc., CA, USA) 1:50 overnight with heat pre-treatment in antigen-retrieval solution, and mouse monoclonal thrombin R (ATAP-2) antibody sc-13503 (Santa Cruz Biotechnology, Inc., CA, USA) 1:20 overnight, with heat pre-treatment in antigen retrieval solution, and antibodies. Rabbit and mouse negative control immunoglobulins (Dako) were used for the negative controls (NC). These markers were evaluated in central (internal perimeter > 6 mm) and distal (internal perimeter ≤ 6 mm) airways. Two independent investigators, using image analysis (Leica microscope, Wetzlar, Germany) at 40× magnification, evaluated the sample immunoreactivity blindly. The length of the basement membrane was evaluated using a Leica Application Suite V3.3 (LAS) software (Leica microscope, Wetzlar, Germany) for Image Analysis. Cell counts were normalized for 1 mm of epithelium, and the results were expressed as the number of positive epithelial cells/mm of the basement membrane, as previously described [40].

### 4.3. Evaluation of Active PAR-1 Immunoreactivity

Thrombin cleaves its receptor in the N-terminal domain between Arg41 and Ser42 [15]. The immunoreactivity of the samples for the active PAR-1 receptor was evaluated using two different antibodies: (1) thrombin R ATAP-2, a mouse monoclonal antibody epitope mapping within the amino acids 42–55; (2) thrombin R H-111, a rabbit polyclonal antibody raised against the amino acid s1–111 of thrombin R of human origin. ATAP-2 antibody recognizes both cleaved and uncleaved receptors, and thrombin R H-111 antibody recognizes the uncleaved receptor but not the cleaved receptor. Therefore, we evaluated the active PAR-1 using the following formula: cells immunoreactive for total PAR-1 (immunostaining with ATAP-2 antibody) minus the immunoreactivity of the cells for uncleaved PAR-1 (immunostaining with H-111 antibody).

### 4.4. Immunofluorescence in Frozen Sections

Frozen sections of the bronchial ring (6 μm) and parenchymal sections (9 μm) were subjected to indirect immunofluorescence as described hereafter. Briefly, the samples were fixed in 4% paraformaldehyde for 15 min at room temperature, followed by three washes in PBS for 3 min each. The samples were then permeabilized with saponin at 0.05% in PBS plus 3% BSA for 5 min at room temperature and, after three washes in PBS, they were fixed in a cold acetone:methanol solution 1:1 at −20 °C for 7 min [40,41]. After the washes in PBS, samples were blocked in 3% BSA in PBS for 1 h at room temperature. Primary antibody incubation was performed in 3% BSA in PBS overnight. The following primary antibodies were used: PAR-1 (ATAP-2) (mouse, 1:20), PAR-1 (H-111) (rabbit, 1:40) antibodies, stored overnight at 4 °C. The next day, slides were washed in PBS, and the secondary antibodies were incubated in 3% BSA in PBS for 1 h at room temperature, followed by three PBS washes. Anti-rabbit IgG (whole molecule)-FITC F7512 (Sigma- Aldrich, Inc., Milan, Italy) and Anti-mouse IgG (whole molecule) R-phycoerythrin conjugate P9287 (Sigma) were used. The samples were then incubated with Hoechst 33,342 (Sigma-Aldrich) 1:1000 in PBS for 10 min at room temperature, followed by a PBS wash. The nuclei were stained with Hoechst 33,342 (#B-2261 SIGMA, Saint Louis, Missouri 63103 USA). The slides are mounted with Vectashield oil (Vector Laboratories, Burlingame, CA, USA), and images were analyzed using a Zeiss laser-scanning microscope at a final magnification of 400×. All immunofluorescence tests were performed with negative controls, where no primary antibody was added.

### 4.5. Cell Cultures

The SV40 large T antigen-transformed cell line from a normal human bronchial epithelial cell line (16HBE) [42], or primary normal human bronchial epithelial (NHBE) cells (ATCC, catalog number PCS-300-010), were used in this study. The source and origin of 16HBE cells were kindly provided by Dr. D. Gruenert Laboratory (University of California, San Francisco, CA, USA) to IBIM CNR, Italy. 16HBE and NHBE cells were cultured as adherent monolayers in Eagle’s minimum essential medium (MEM) supplemented with 10% heat-inactivated (56 °C, 30 min) fetal bovine serum (FBS), 1% MEM (non-essential aminoacids, Euroclone), 2 mM L-glutamine and gentamicin 250L g/mL at 37 °C in a humidified 5% CO^2^ atmosphere [40].

### 4.6. Preparation of Cigarette Smoke Extracts (CSE)

Commercially available cigarettes (Marlboro Red Label, Philip Morris International, Switzerland) were used in this study. CSE was prepared and further diluted to the required concentrations in fresh culture medium, as previously described [43]. Each cigarette was smoked for 5 min, and two cigarettes were used per 50 mL of PBS to generate a CSE–PBS solution. The CSE–PBS solution was filtered through a 0.22-µm pore sieve to remove bacteria and large particulates. The smoke solution was then adjusted to pH 7.4. To prevent the possible inactivation of residues present in the filtered CSE–PBS solution, it was kept in the dark and used within 30 min of preparation. This solution was considered as a 100% CSE and subsequently diluted to obtain the desired concentrations in each experiment. The concentration of CSE was calculated spectrophotometrically, measuring the OD at between 270 and 280 nm. The pattern of absorbance among different batches showed very low differences. The concentration, expressed as arbitrary units per milliliter, was calculated based on the following formula: ODmax × 2 × dilution factor. The CSE was further diluted to the required concentration in fresh culture medium. Furthermore, to exclude the toxic effect of CSE 10%, the viability of the cells exposed to CSE was evaluated with a trypan blue exclusion dye assay. Cigarette smoke extract was used to stimulate cultured 16-HBE and NHBE cells lines.

### 4.7. Stimulation of the Cells

16HBE (100,000 cells/well) and NHBE (200,000 cells/well) were plated in standard six-well plates, in a suitable medium at 10% FBS, and grown to confluence (70–80%). After this, the medium was removed and replaced with an FBS-free complete medium for an additional 4 h (5% CO_2_ at 37 °C) to make the cells quiescent. Subsequently, the medium was replaced with 1% FBS cells, which were stimulated with TR 2, at 5 U/mL (Cod. T4393 Sigma-Aldrich, Milan, Italy) in the presence or absence of CSE (10%) for 24 h to study active PAR-1, for 4 h to study cell apoptosis, and for 72 h to study cell proliferation. At the end of treatment, the cells were collected using a cell scraper for flow cytometric analysis. Finally, the cells were stimulated with TR 2.5 U/mL (Cod. T4393 Sigma-Aldrich, Milan, Italy) in the presence or absence of CSE (10%) and SFLLRN-NH2 (50 μM), a PAR-1 selective activating peptide (S1820 Sigma-Aldrich, Milan, Italy), for 24 h to study IL-8 release in the cell culture supernatants.

### 4.8. Flow-Cytometry Analyses in 16HBE Cell Line

The quantitative expression of PAR-1 (ATAP-2) and PAR-1 (H-111) was determined in 16HBE cells by flow cytometry analyses using indirect label immunofluorescence with a FACS Calibur™ flow cytometer (Becton Dickinson, Mountain View, CA, USA) supported by Cell Quest acquisition and data analysis software (Becton Dickinson, Mountain View, CA, USA), as previously described [40]. Cells were grown until 70–80% of confluence in the presence or absence of CSE 10% for 24 h, thrombin 2.5 U/mL for 1 h, or in combination as described earlier. After harvesting, cells were detached from the six-well plates with a cell scraper, washed in PBS, and collected in FACS tubes. Afterward, cells were washed in staining buffer (PBS containing 1% FCS and 0.1% NaN_3_) and then fixed with 4% paraformaldehyde in PBS, washed again, and permeabilized with PBS (containing 0.1% saponin, 1% sodium azide, and 10% FBS). Then, the cells were incubated with the primary antibodies anti-PAR-1R (ATAP-2) (sc-13503, Santa Cruz Biotechnology, Inc., CA, USA) and with rabbit polyclonal anti-PAR-1 (H-111) (sc-5605, Santa Cruz Biotechnology, Inc., CA, USA) for 1 h at 4 °C to evaluate their expression. FITC-conjugated polyclonal swine anti-rabbit Ig (DAKO Glostrup, Denmark), mouse IgG (whole-molecule) Ab R-phycoerythrin conjugate (P9287, Sigma) were used as secondary antibodies in the dark for 1 h at RT, respectively, for anti-PAR-1 (H-111) and for anti-PAR-1 (ATAP-2). The unbound antibodies were washed with cold PBS, and fluorescence-positive cells were quantified by flow cytometer analysis. The percentages of positive cells for PAR-1 (ATAP-2) and PAR-1 (H-111) were determined from forward (FS) and sideways (SS) scatter patterns, after gating on the cells, excluding debris. Cells were considered positive if their fluorescence 1 (FL1) was greater than a gate set to exclude all cells in the FL1 peak from an isotype-matched control antibody [44]. Non-specific binding and background fluorescence were quantified by analyzing the negative control. The results of flow cytometry were expressed as the percentage total of positive cells. Active PAR-1 was calculated as previously described.

### 4.9. Immunofluorescence in 16HBE and NHBE Cells

16HBE cells lines were seeded in six-well plates within which we inserted a sterile cover slide. Cells were grown until 70–80% of confluence in the presence or absence of CSE for 24 h, thrombin 2.5 U/mL for 1 hr, or in combination, and the cover slide was recovered. The cells were fixed in paraformaldehyde at 4% 15 min. RT, washed in PBS and treated for 5 min with permeabilization buffer (0.05% saponin in PBS plus 3% BSA), fixed in cold acetone, and then incubated with blocking solution (BSA 0.5% in PBS) for 1 h RT. Immunofluorescent staining was performed with the rabbit polyclonal antibody PAR-1 (H-111) (Santa Cruz Biotechnology) 1:40 overnight, and the monoclonal mouse PAR-1 (ATAP-2) 1:25 overnight, all at 4 °C; all antibodies, diluted in PBS plus 3% BSA, were incubated overnight at 4 °C. Non-immune IgG was used as a negative control. Secondary antibodies, anti-rabbit IgG-FITC (whole molecule) (F7512, Sigma-Aldrich, Milan Italy), Milan Italy, and anti-mouse IgG (whole-molecule) R-phycoerythrin conjugate (P9287 Sigma), were stored in the dark for 1 h at room temperature. After washing in PBS, the nuclei were counterstained with Hoechst 33,342 (Sigma–Aldrich) 1:1000 in PBS for 10 min at room temperature, followed by washing in PBS. The slides were mounted on Vectashields (Vector Laboratories, Burlingame, CA, USA). The images were analyzed using a Zeiss laser scanning microscope (Zeiss, Göttingen, Germany) at a final magnification of 400x40].

### 4.10. Laser Capture Microdissection

Laser capture microdissection (LMD) was performed using the Leica AS LMD (Leica Microsystems, Wetzlar, Germany) from three no-smoking COPD and three smoking COPD patients. Epithelial cells (recognized by their morphological characteristics) were micro-dissected from the sample into the cap of a microtube and then processed in the same tube to extract the mRNA. Normal human bronchial epithelial cells (16HBE) were used to detect the normal value of the markers, as previously described [40].

### 4.11. RNA Isolation and qRT-PCR

Total RNA was extracted from 16-HBE and from the epithelium of COPD smokers and COPD Ex-smokers, micro-dissecting tissues according to the method used by Chomczynski and Sacchi (1987), as previously described [45], with TRIzol Reagent (Invitrogen, CA, USA) following the manufacturer’s instructions. It was then reverse-transcribed into cDNA, using M-MLV-RT and oligo(dT) primer (Invitrogen, CA, USA). Quantitative real-time PCR PAR-1 gene transcription was carried out on an ABI PRISM7900 HT Sequence Detection Systems (Applied Biosystems, Foster City, CA, USA) using specific FAM-labeled probes and primers. Gene expression was normalized to the glyceraldehyde-3-phosphate dehydrogenase (GAPDH) endogenous control gene. The relative quantitation of gene expression was carried out using the comparative CT method (2^−ΔΔ*C*t^) and was plotted as a fold-change compared to 16HBE, chosen as the reference sample.

### 4.12. Cell Apoptosis

The cells were stained with a solution containing a mixture of annexin V-FITC in binding buffer 1x After incubation (15 min in total darkness, RT), we added propidium iodide just before the analysis. The number of viable, apoptotic cells was determined by cytofluorimetry (FACS Calibur, Becton Dickinson, Mountain View, CA, USA). The results were presented as a fold change of the total percentage above untreated cells ± SD. The method of cell apoptosis was previously described in [46].

### 4.13. Cell Proliferation Assay

Cell proliferation was measured using a carboxyfluorescein succinimidyl ester (CFSE) labeling assay. CFSE is used to fluorescently label live cells and is equally partitioned to daughter cells during division. The method was previously described in the study by [46]. Briefly, the cells were incubated with CFSE (Molecular Probes, Inc. Eugene, OR) (at a final concentration of 5 μM) at 37 °C for 10 min. Labeling was blocked by the addition of an equal volume of heat-inactivated FCS. The tubes were placed in ice for 5 min and then washed. The cells were plated at 1 × 10^5^ cells/well in six-well plates and incubated at 37 °C with 5% CO_2_. The cells were harvested with FBS for 24 h and then stimulated as described earlier. Cell proliferation was assessed by flow cytometry.

### 4.14. Measurements of IL-8 Release

The levels of IL-8 were determined in NHBE supernatants using a commercial ELISA kit (R&D Systems, Inc., MN, USA), according to the manufactures’ specifications. The lower detection limit for IL-8 was < 5 pg/mL.

## 5. Statistical Analysis

Statistical comparisons to test the differences between the two groups (HC, COPD) were made using an unpaired *t*-test (made by using the Kruskal-Wallis test followed by Fisher’s PLSD correction for multiple comparisons). Data were expressed as median and interquartile ranges. An ANOVA test with Fisher’s correction for multiple comparisons or Student’s *t*-test were used for the analysis of the data obtained from in vitro experimental conditions. Data were expressed as mean ± SD. Correlation analyses were performed using Spearman’s rank test. All statistical analyses were performed using Stat View^®^ 5 software (SAS Institute, Inc.). A *p*-value of less than 0.05 was considered to indicate statistical significance in these analyses.

## 6. Conclusions

In conclusion, our study demonstrated that PAR-1 is present in epithelial cells from the central and distal airways of COPD patients and is present at higher levels in central airways, mainly being affected by cigarette smoke. The evaluation of active PAR-1 in both central and distal airways described a significant increase in COPD compared to the HC, with higher values in COPD patients who smoke. All these findings support the idea that cigarette smoke promotes the expression and activity of PAR-1 during the mechanism of airway inflammation, and potentially affects the progressive decline in lung function. However, due to the limited sample size of the patients in our study, it is difficult to conclude and establish a definite outcome. Further studies might be necessary to underline the descriptive nature of our findings, and of the role of PAR-1 in patients with different grades of severity of COPD. Accordingly, we suggest delving into certain mechanistic aspects of the lung physiology associated with PAR-1, e.g., epithelial barrier integrity. However, our study might be of significant impact for agonists/antagonists of PARs, representing a therapeutic strategy for the treatment of certain respiratory diseases that include COPD.

## Figures and Tables

**Table 1:** Demographic characteristics of the subjects.

	HC (*n* = 10)	COPD (*n* = 15)
**Gender (M/F)**	7/3	10/5
**Smoke**	-	9/6
**Age (years)**	66.5 ± 6.5	63 ± 8.1
**Pack/Years**	-	41.8 ± 28.5
**FEV1 % of predicted**	89 ± 16	74 ± 17.9
**FEV1/FVC of predicted**	83 ± 6	67 ± 10.6
